# Bid chimeras indicate that most BH3-only proteins can directly activate Bak and Bax, and show no preference for Bak *versus* Bax

**DOI:** 10.1038/cddis.2015.105

**Published:** 2015-04-23

**Authors:** C Hockings, K Anwari, R L Ninnis, J Brouwer, M O'Hely, M Evangelista, M G Hinds, P E Czabotar, E F Lee, W D Fairlie, G Dewson, R M Kluck

**Affiliations:** 1 The Walter and Eliza Hall Institute of Medical Research, 1G Royal Parade, Parkville, Victoria 3052, Australia; 2 Department of Medical Biology, The University of Melbourne, Parkville, Victoria 3010, Australia; 3 School of Chemistry and Bio21 Molecular Science and Biotechnology Institute, University of Melbourne, Parkville, Victoria 3010, Australia

## Abstract

The mitochondrial pathway of apoptosis is initiated by Bcl-2 homology region 3 (BH3)-only members of the Bcl-2 protein family. On upregulation or activation, certain BH3-only proteins can directly bind and activate Bak and Bax to induce conformation change, oligomerization and pore formation in mitochondria. BH3-only proteins, with the exception of Bid, are intrinsically disordered and therefore, functional studies often utilize peptides based on just their BH3 domains. However, these reagents do not possess the hydrophobic membrane targeting domains found on the native BH3-only molecule. To generate each BH3-only protein as a recombinant protein that could efficiently target mitochondria, we developed recombinant Bid chimeras in which the BH3 domain was replaced with that of other BH3-only proteins (Bim, Puma, Noxa, Bad, Bmf, Bik and Hrk). The chimeras were stable following purification, and each immunoprecipitated with full-length Bcl-x_L_ according to the specificity reported for the related BH3 peptide. When tested for activation of Bak and Bax in mitochondrial permeabilization assays, Bid chimeras were ~1000-fold more effective than the related BH3 peptides. BH3 sequences from Bid and Bim were the strongest activators, followed by Puma, Hrk, Bmf and Bik, while Bad and Noxa were not activators. Notably, chimeras and peptides showed no apparent preference for activating Bak or Bax. In addition, within the BH3 domain, the h0 position recently found to be important for Bax activation, was important also for Bak activation. Together, our data with full-length proteins indicate that most BH3-only proteins can directly activate both Bak and Bax.

The Bcl-2 family of proteins controls the mitochondrial pathway of apoptosis, a process often dysregulated in cancer and other diseases.^1, 2, 3^ Apoptotic triggers including DNA damage and oncogene activation cause the synthesis or activation of one or more pro-apoptotic Bcl-2 homology region 3 (BH3)-only proteins,^1, 2, 3, 4^ a subfamily that includes Bid, Bim, Puma, Noxa, Bad, Bik, Bmf and Hrk. These proteins then engage via their BH3 domain with other Bcl-2 family members. BH3-only proteins that can directly bind and activate the Bcl-2 effector proteins Bak or Bax are called ‘activators'.^5^ When Bak or Bax become activated and oligomerize in the mitochondrial outer membrane (MOM), the apoptotic ‘switch' has flipped and the cell is committed to cell death. The prosurvival members (Bcl-2, Bcl-x_L_, Mcl-1, Bcl-w, Bfl-1/A1 and Bcl-B) inhibit apoptosis by specifically binding both the BH3-only proteins and activated Bak and Bax.^6, 7, 8, 9, 10, 11^ Thus, the cell's complement of prosurvival proteins, Bak, and Bax, determines the sensitivity of that cell to each BH3-only protein, and by extension to each type of pro-apoptotic stimulus.

A thorough understanding of BH3-only proteins is crucial for the development of cancer therapeutics such as the new class of anti-cancer molecules called BH3 mimetics that are showing significant promise in clinical trials.^12, 13^ The binding of BH3-only proteins to prosurvival proteins has been well-characterized and revealed significant preferences for engaging different members.^6, 8, 9^ How BH3-only proteins bind and activate Bak and Bax remains less understood for several reasons. First, generating stable recombinant BH3-only proteins is difficult because, except for Bid, they are intrinsically disordered^14, 15, 16^ and because most contain hydrophobic C-terminal membrane anchors.^17^ Thus, most *in vitro* studies of BH3-only proteins have used synthetic peptides corresponding to the BH3 domains, C-terminally truncated recombinant proteins or *in vitro* translated (IVT) proteins. Second, BH3-only reagents bind poorly to recombinant Bak and Bax in the absence of membranes, although detergents and liposomes may substitute for the MOM.^18, 19, 20^ Third, activation of Bak and Bax on mitochondria can be complicated by the presence of other proteins such as prosurvival proteins. Indeed, genetically altering BH3-only protein levels in mice resulted in complex phenotypes due to multiple interactions between family members, precluding firm conclusions as to which BH3-only proteins are direct activators.^18, 21, 22^

Bid and Bim are direct activators according to a variety of approaches,^5, 8, 9, 23, 24^ and were recently proposed to be specific for Bak and Bax, respectively.^25^ Early studies using Noxa BH3 peptides^5, 8^ and IVT Noxa^9^ concluded that Noxa was not an activator. However, in more recent studies a Noxa BH3 peptide^23^ and purified recombinant NoxaΔC^20^ were found to be activators of both Bak and Bax. Puma has also been described as both an activator^26, 27^ and not an activator.^8, 28^ Du *et al.*^23^ analyzed the full panel of BH3 peptides and classified Bim as a strong activator, Bid, Noxa and Bmf as moderate activators, and Puma, Bik and Hrk as weak activators. The only BH3-only member that has never been described as an activator is Bad.

While BH3 peptides and recombinant truncated BH3-only proteins have been useful for *in vitro* studies, new reagents that target mitochondria may better reflect the behavior of the parent proteins. As Bid is stable as a recombinant protein, we generated chimeras of Bid in which the BH3 domain of Bid was replaced with that of seven other BH3-only proteins. This is a similar approach to the Bim chimeras used for expression in cells^18^ and in mice.^29^ More recently, truncated Bid (tBid) chimeras containing the BH3 domains of Bim, Bak and Bax as well as those of the prosurvival proteins, have been generated as IVT proteins.^11^

To compare the ability of BH3-only proteins to activate Bak and Bax *in vitro*, we incubated Bid chimeras and BH3 peptides with mitochondria containing either Bak or Bax. We found that the membrane-targeted Bid chimeras were much more potent activators than their related BH3 peptides, and that all BH3 domains except for Bad and Noxa were activators to some extent. We conclude that activation of Bak and Bax may be underestimated by studies using BH3 peptides, and that even BH3-only proteins such as Bik, Bmf and Hrk that are often considered unable to activate Bak or Bax, may act as activators under certain conditions.

## Results

### Bid BH3 chimeras are stable as recombinant proteins

**Figure 1:**
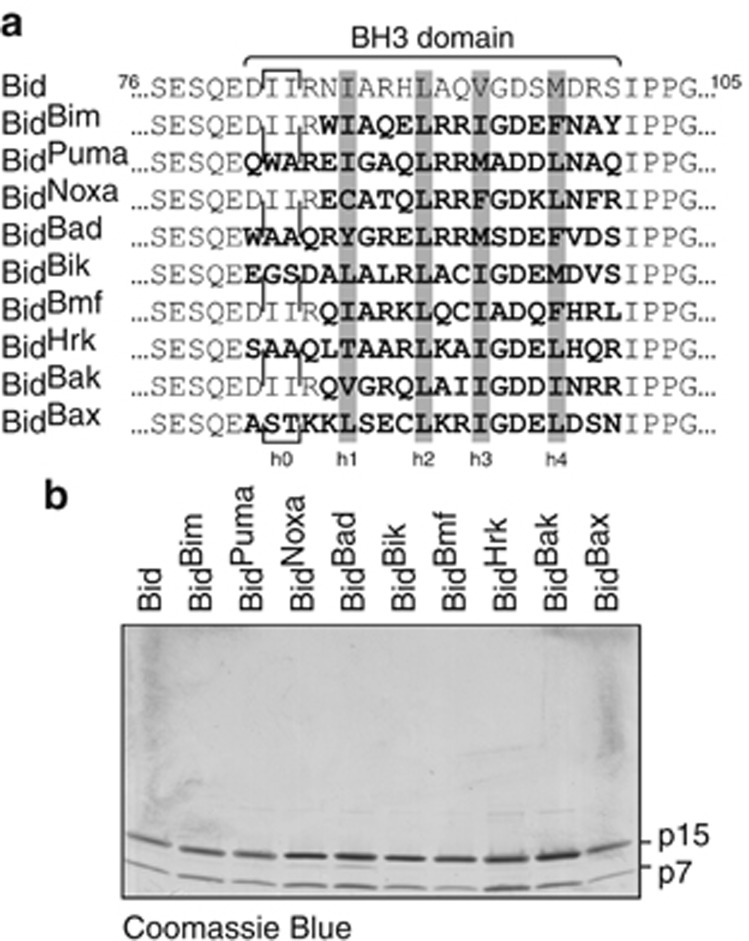
Bid BH3 chimeras are stable recombinant proteins. (**a**) Sequence of Bid BH3 chimeras. Sequence alignment shows the Bid BH3 domain swapped for that of other Bcl-2 family members. Hydrophobic residues (h1–h4) that are important for binding to prosurvival proteins^31^ are highlighted, as are the recently identified h0 residues that are important for binding to Bax.^24^ (**b**) Recombinant Bid BH3 chimeras. Chimeras were cleaved by thrombin during purification to generate N-terminal p7 and C-terminal p15 fragments of Bid.^30^ Chimeras were separated by SDS-PAGE and stained for total protein

Bid is the only BH3-only protein that can be readily expressed and purified as a recombinant protein. To generate stable proteins that mimic the behavior of the other BH3-only proteins the BH3 domain of human Bid (residues 81–100) was replaced with the BH3 domains of 7 other human BH3-only proteins to create the Bid^Bim^, Bid^Puma^, Bid^Noxa^, Bid^Bad^, Bid^Bik^, Bid^Bmf^ and Bid^Hrk^ chimeras (Figure 1a). Bid chimeras containing the BH3 domains of Bak and Bax were also generated to explore Bak and Bax function, and to allow comparison with the tBid^Bax^ and Bid^Bak^ chimeras reported previously.^11^ To simplify purification, the caspase-8 cleavage site in Bid was replaced with a thrombin cleavage site, allowing a combined cleavage/purification step.^30^ Thus, all Bid chimeras presented in this study are ‘cleaved' Bid (also called n/cBid or p7/p15 Bid). A C-terminal haemagglutinin (HA) tag was added to detect chimeras by immunoblotting, and shown not to affect function (Supplementary Figure 1). The swapped BH3 region was initially 20 residues covering the 4 hydrophobic residues (h1–h4) important for binding to prosurvival proteins^31^ and the 2 hydrophobic residues (h0) recently shown to be important for binding to Bax.^24^ However, four of the chimeras (those with Bim, Noxa, Bmf and Bak BH3 domains) expressed poorly or aggregated during purification (not shown). This may be due to residues that correspond to I83 in Bid (glutamate from Bim, Noxa and Bmf or methionine from Bak, Supplementary Figure 2a) being poorly tolerated. I83 normally makes contact with four hydrophobic residues in helices 1 and 8 that may stabilize the protein (Supplementary Figure 2b).^32^ Accordingly, when the swapped domain was shortened to 16 residues to retain I83, the 4 chimeras were stable (Figure 1b). As expected, the p15 fragments of all Bid chimeras retained the ability to translocate to membranes of mouse liver mitochondria (MLM) (Supplementary Figure 2c).

### Bid BH3 chimeras exhibit specific binding to prosurvival proteins

**Figure 2:**
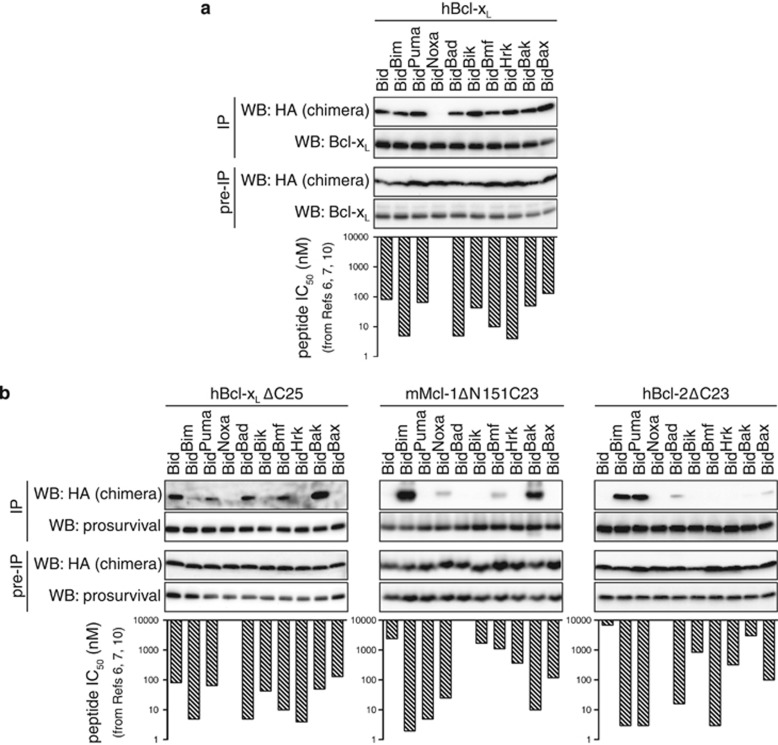
Bid BH3 chimeras show specific binding to prosurvival proteins. (**a**) Bid BH3 chimeras show specificity in binding to full-length Bcl-x_L_. Bid BH3 chimeras and full-length Bcl-x_L_ were incubated at a 1 : 1 molar ratio in the presence of *Bak*^−/−^ MLM before solubilization with 1% digitonin, immunoprecipitation (IP) of Bcl-x_L_ and Western blotting for bound chimera (HA tag). (**b**) Bid BH3 chimeras binding to truncated Bcl-x_L_, Mcl-1 and Bcl-2. The chimeras were incubated as in (**a**) with the indicated truncated prosurvival proteins. Blots are representative of three independent experiments (shown in Supplementary Figure 3a). Bottom panels show previously published IC_50_ values of the equivalent BH3 peptides for binding to truncated prosurvival proteins as measured by SPR.^6, 7, 10^

To evaluate the Bid BH3 chimeras, they were first tested for binding to the Bcl-x_L_ prosurvival protein (Figure 2a and Supplementary Figure 3a). Equimolar chimera and full-length Bcl-x_L_ were incubated in the presence of *Bak*^−/−^ MLM, and the immunoprecipitated Bcl-x_L_ examined for bound chimera. Each chimera, except for Bid^Noxa^, co-precipitated strongly with Bcl-x_L_ (Figure 2a, upper panel), consistent with the binding of human BH3 peptides to Bcl-x_L_ΔC25 measured by surface plasmon resonance (SPR) (Figure 2a, lower panel).^6, 7, 10^

### C-terminally truncated prosurvival proteins have altered BH3-binding specificity when tested on mitochondria

When C-terminally truncated Bcl-x_L_ (ΔC) was tested for binding to Bid chimeras its specificity was significantly different to full-length Bcl-x_L_, with four chimeras (Bid^Bim^, Bid^Bik^, Bid^Hrk^ and Bid^Bax^) showing little co-precipitation (Figure 2b and Supplementary Figure 3a). Chimeras bound to the canonical hydrophobic groove in full-length Bcl-x_L_, rather than a site involving the C terminus, as the BH3 mimetic ABT-737 prevented co-precipitation (Supplementary Figure 3b). Altered binding may be due in part to truncated Bcl-x_L_ remaining in the supernatant, whereas full-length Bcl-x_L_ (and Bid chimeras) was predominantly membrane associated (Supplementary Figure 3c). Consequently, the hydrophobic groove may adopt a different conformation and therefore binding specificity on membrane insertion.

The chimeras also showed specific binding to truncated Mcl-1 (ΔNΔC) and to truncated Bcl-2 (ΔC) (Figure 2b and Supplementary Figure 3a). While binding was similar to that of BH3 peptides on SPR, there were some exceptions. For example, Bid^Puma^ bound poorly to Mcl-1ΔNΔC and Bid^Bmf^ bound poorly to Bcl-2ΔC (Figure 2b). Thus, membrane insertion may alter the binding profile of several prosurvival proteins.

In the absence of mitochondria, the chimeras immunoprecipitated only weakly with truncated prosurvival proteins (data not shown), consistent with the requirement of membranes for separation of the Bid p7 and p15 fragments and exposure of the Bid BH3 domain.^33, 34, 35^ Accordingly, SPR (which is performed in the absence of membranes) also failed to detect binding of the chimeras to Bcl-x_L_ΔC and Mcl-1ΔNΔC even in the presence of octyl glucoside (data not shown), a detergent commonly used to separate the p7 and p15 fragments of Bid.^36^

In summary, binding of the chimeras to full-length Bcl-x_L_ (in the presence of mitochondria) reflected the specificity of their cognate BH3 peptide. In addition, the importance of studying full-length Bcl-2 proteins in their native environments was highlighted by the altered binding specificity of Bid chimeras to truncated prosurvival proteins.

### Most BH3 chimeras and peptides activate Bak and Bax, but with different potencies

To compare the ability of each chimera or related BH3 peptide to activate Bak, wild-type C57BL/6 MLM were incubated with increasing concentrations of chimera or BH3 peptide and tested for cytochrome *c* release. To test activation of Bax, MLM from *Bak*^−/−^ mice were supplemented with recombinant full-length Bax.^37^ MLM have been used previously to assess Bak and Bax activation^11, 24, 38, 39^ as the remaining Bcl-2 proteins were undetectable except for Bcl-x_L_ which was present at low levels (<3 nM).^40^ Cytochrome *c* release was used as a measure of Bak or Bax activation as the two events correlated strongly in wild-type MLM (Supplementary Figure 1), and no cytochrome *c* release occurred in *Bak*^−/−^ MLM without addition of Bax (data not shown).^5, 40, 41^

**Figure 3:**
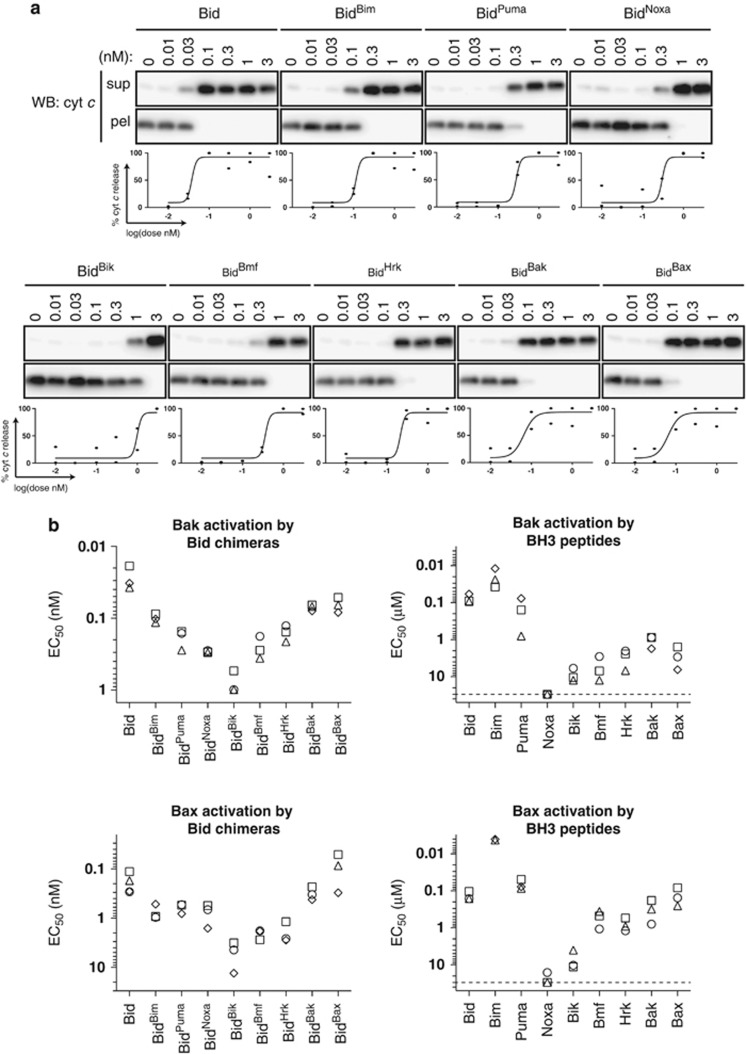
Bid chimeras and BH3 peptides are similar in their specificity for activating both Bak and Bax. (**a**) Bid chimeras activate Bak to release cytochrome *c*. Wild-type MLM were treated with the indicated Bid chimeras and supernatant (sup) and pellet (pel) fractions Western blotted for cytochrome *c* (top panels). Quantification of cytochrome *c* release allowed estimation of EC_50_ (bottom panels). (**b**) Comparison of Bak and Bax activation by Bid chimeras and BH3 peptides. Wild-type MLM (Bak activation) or *Bak*^−/−^ MLM plus 10 nM Bax (Bax activation) were treated with Bid chimeras or BH3 peptide. Cytochrome *c* release was quantified as in **a**. The EC_50_ values from three or four independent experiments are shown, and in each graph the values from one experiment are represented by the same symbol. Note that EC_50_ values for Bid^Bad^ and Bad peptide were not estimated because cytochrome *c* release was often incomplete (see Figure 4)

Most Bid chimeras induced dose-dependent activation of Bak (release of cytochrome *c*), allowing estimation of their EC_50_ (Figure 3a). Similar experiments performed for Bak activation by BH3 peptides, and for Bax activation by Bid chimeras and peptides, allowed us to compare Bak and Bax activation by both types of reagents (Figure 3b). Bid and Bid^Bim^ and the related peptides were strong activators of both Bak and Bax (Figure 3b), consistent with previous reports.^5, 9, 23^ Bid^Puma^ chimera and Puma peptide were also activators, in agreement with some^9, 23, 26, 27^ but not other reports.^8, 28^ Bmf, Hrk and Bik reagents were activators, with Bik being the weakest. While Bid^Noxa^ could also activate both Bak and Bax, the Noxa peptide had no apparent activator activity. As Bid^Bad^ chimera and Bad peptide often failed to completely release cytochrome *c*, the EC_50_ was not included in Figure 3b. The Noxa and Bad results are discussed in more detail below. Finally, Bid^Bak^ and Bid^Bax^ chimeras (and equivalent BH3 domain peptides) could activate both Bak and Bax, as previously reported,^11^ and consistent with Bak and Bax being able to auto-activate.^42^

By comparing both Bak and Bax activation by each chimera and BH3 peptide, we could draw several conclusions. First, the Bid chimeras were generally >1000-fold more potent than their related BH3 peptide in activating Bak. This was attributable to the Bid scaffold targeting all chimeras to the MOM (Supplementary Figure 2c), consistent with targeting a Bid peptide to liposomes making it nearly as potent as the Bid protein in activating Bax.^43^ The Bid scaffold may also increase the affinity of the BH3 domain by stabilizing its structure, or by aligning the BH3 domain to the activation site on Bak and Bax. Second, the chimeras are reasonable mimics of their parent BH3-only proteins, as the relative potency of each chimera, with the exception of Noxa, reflected the relative potency of the cognate BH3 peptide (Figure 3b). Third, the relative potencies of the chimeras for activating Bak and Bax were similar, as were the potencies of the peptides (Figure 3b and Supplementary Figure 4). Thus, while BH3-only proteins show specificity for certain prosurvival proteins, they may not for Bak and Bax. In particular, in contrast to a recent report,^25^ there was little evidence of Bid preferentially targeting Bak and Bim preferentially targeting Bax, as Bid^Bim^ was approximately fourfold less potent than Bid in activating both Bak and Bax.

### Bad does not directly activate Bak or Bax, but can do so indirectly

**Figure 4:**
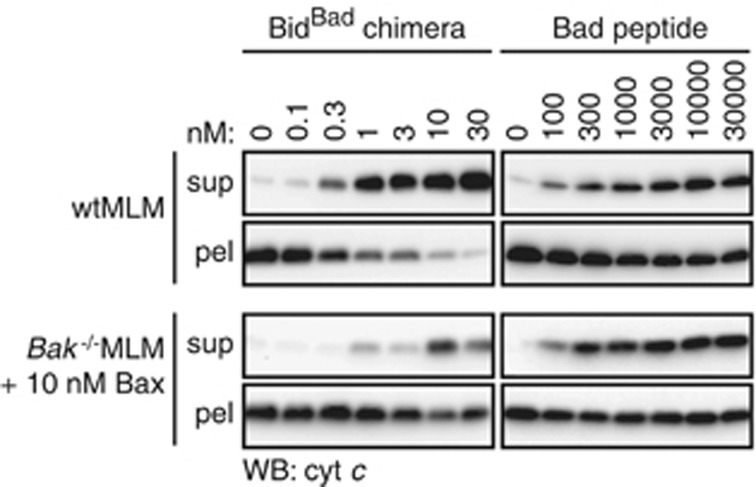
Bad-like reagents often induce only partial cytochrome *c* release. Wild-type MLM or *Bak*^−/−^ MLM plus 10 nM Bax were treated with Bid^Bad^ or Bad peptide as in Figure 3 and assessed for cytochrome *c* release. Blots are representative of at least four independent experiments (shown in Supplementary Figure 5)

Compared with the other BH3-only reagents, the Bid^Bad^ chimera and Bad peptide caused a more graded and often incomplete release of cytochrome *c,* particularly in the Bax experiments (Figure 4 and Supplementary Figure 5). This made EC_50_ estimations problematic, and suggested that the Bad-like reagents caused cytochrome *c* release indirectly (also called de-repression). As Noxa peptide, that does not bind to Bcl-x_L_,^6^ did not cause any cytochrome *c* release (Figure 3b), it is possible that the Bad-like reagents bind endogenous Bcl-x_L_ present on MLM to liberate endogenous activators that then activate Bak or Bax. Variable cytochrome *c* release induced by Bad-like reagents (Supplementary Figure 5) might be explained by variation in the levels of Bcl-x_L_ and/or direct activators between mitochondria preparations.

**Figure 5:**
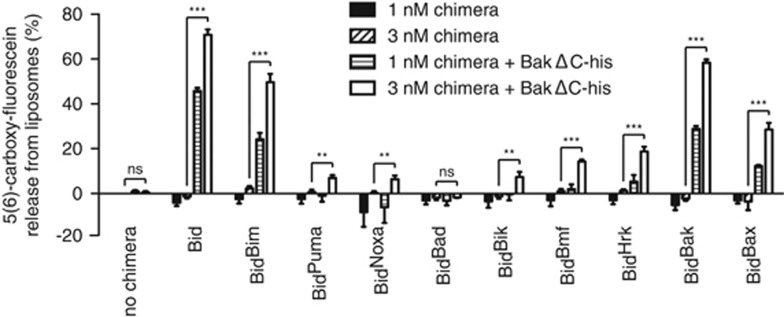
Each Bid chimera, except for Bid^Bad^, can activate recombinant Bak to permeabilize liposomes. Nickel-chelating liposomes were treated with 150 nM His-tagged BakΔC and Bid chimeras as indicated and tested for the release of 5(6)-carboxy-fluorescein. Data were normalized by subtracting the signal from untreated liposomes, and then expressed as a percentage of the signal induced by permeabilization with 1% CHAPS. Error bars indicate the S.E.M. of four separate experiments. Statistical significance (‘NS' not significant, **P<0.01, ***P<0.001) was calculated using non-normalized data and a one-tailed Student's *t*-test

To further test direct activation by the chimeras, they were tested for their ability to activate His-tagged BakΔCT on nickel-chelating liposomes (Figure 5), as described previously.^44^ While the dose range (from 1 to 3 nM) was more limited in this assay to avoid non-specific permeabilization, each chimera except for Bid^Bad^ was able to activate Bak and permeabilize the liposomes. Moreover, the activation profile matched that for full-length Bak in MLM (Figures 3a and b), indicating that other Bcl-2 family proteins in MLM probably had little impact on cytochrome *c* release by all chimeras except Bid^Bad^.

### Noxa does not directly activate Bak or Bax

**Figure 6:**
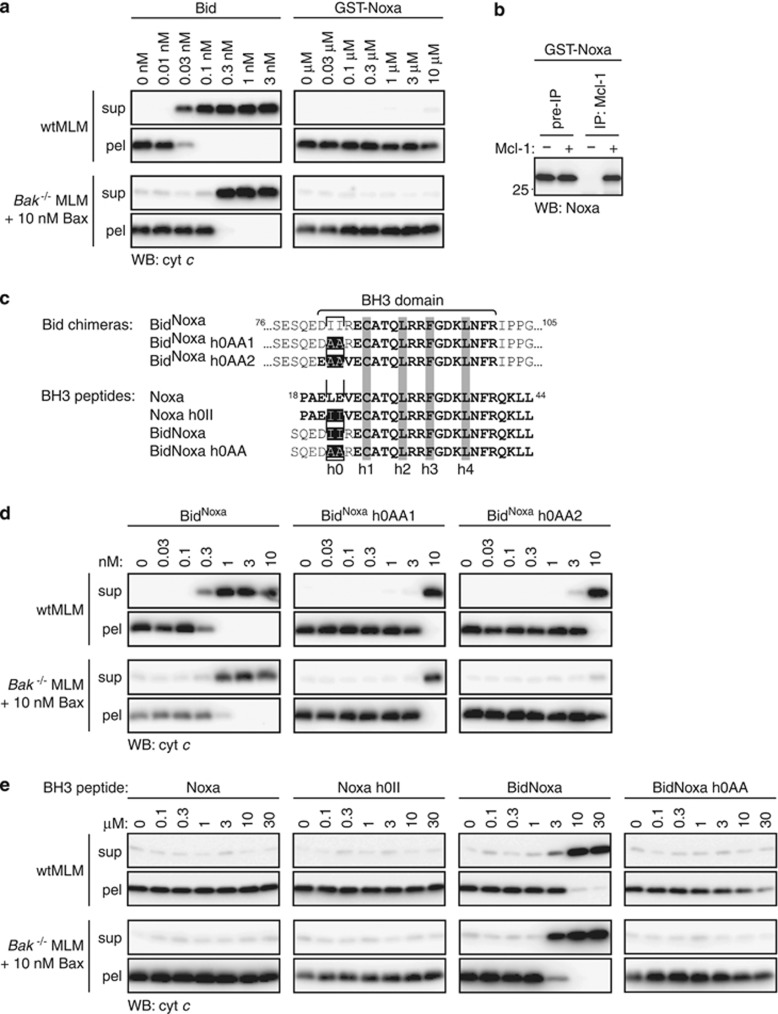
Noxa is not a direct activator of Bak or Bax. (**a**) GST-NoxaΔC does not activate Bak or Bax. Wild-type MLM or *Bak*^−/−^ MLM plus 10 nM Bax were treated with the indicated Noxa reagents and tested for cytochrome *c* release. (**b**) GST-NoxaΔC binds to Mcl-1. GST-NoxaΔC was incubated with Mcl-1ΔNΔC at a 1 : 1 molar ratio in the presence of *Bak*^−/−^ MLM before immunoprecipitation of Mcl-1 and Western blotting for Noxa. (**c**) Sequences of Bid^Noxa^ chimera and Noxa BH3 peptide mutations. (**d**) Activator function of Bid^Noxa^ depends partially on the h0 residues of Bid. The Bid^Noxa^ chimera variants were tested for activator function as in **a**. (**e**) Noxa peptide conversion to an activator depends on the h0 residues of Bid. The Noxa BH3 peptide variants were tested for activator function as in **a**. Blots are representative of two or more independent experiments

The finding that Bid^Noxa^, but not Noxa peptide, could activate Bak and Bax (Figure 3) prompted us to test a recombinant variant of the Noxa protein (Figure 6a). GST-NoxaΔC contains GST at the N-terminus for increased stability and lacks the C-terminal membrane anchor, similar to a variant of Noxa (S peptide-NoxaΔC) reported to bind and activate BakΔC in liposomes.^20^ In the MLM, even very high concentrations (10 *μ*M) of GST-NoxaΔC did not activate Bak or Bax (Figure 6a) despite the Noxa BH3 domain being available for binding to Mcl-1 (Figure 6b).

### The Bid h0 contributes to Bak and Bax activation

Mutagenesis was then used to examine why the Bid^Noxa^ chimera activated Bak and Bax, but Noxa peptide and GST-NoxaΔC did not (Figures 3b and 6a). As noted above, the chimera contains the Bid sequence (QEDIIR) around the h0 region recently found to be important for activating Bax.^24^ To test whether the h0 region might also contribute to Bak activation, and account for the activator activity of Bid^Noxa^, we replaced the two h0 residues in Bid^Noxa^ with alanine, flanked by residues from Bid (Bid^Noxa^h0AA1) or from Noxa (Bid^Noxa^h0AA2) (Figure 6c). Activation of both Bak and Bax was decreased by ~10-fold (Figure 6d), indicating that h0 residues contribute to activation of Bak as well as Bax. In the reverse approach, we attempted to convert the Noxa peptide to an activator by adding Bid sequence (Figure 6c). Introducing just the two h0 isoleucines of Bid (Noxa h0II peptide) was not sufficient, but introducing seven Bid residues (BidNoxa peptide) to mimic the swap in the Bid^Noxa^ chimera now converted the peptide to an activator (Figure 6e). Within those seven Bid residues, the h0 residues were required as their substitution with alanine (BidNoxa h0AA peptide) abrogated Bak activation (Figure 6e). Residues flanking h0 may also promote or hinder activation, as BidNoxa peptide but not Noxa h0II peptide could activate Bak and Bax (Figure 6e) and the Bid^Noxa^ h0AA1 and hOAA2 mutant chimeras did not lose all activator function (Figure 6d). Results with the Noxa peptide are consistent with reduced Bak activation by a Bid peptide in which truncation had removed the h0 region.^39^ In summary, these experiments show that h0 in both peptide and chimera can contribute to Bak activation, as well as Bax activation.^24^

## Discussion

The Bid chimeras were generally >1000 times more potent than BH3 peptides at directly activating Bax/Bak (Figure 3), as previously shown for Bid protein *versus* Bid BH3 peptide.^8, 43^ The potency of the chimeras may be largely due to membrane targeting by the Bid scaffold, as targeting a Bid peptide to mitochondria rendered it almost as potent as Bid protein;^43, 45^ induced helicity of the BH3 domain^46, 47^ or improved ‘presentation' of the BH3 domain to Bak or Bax may also contribute. As all BH3-only proteins (except Bad) contain a C-terminal mitochondrial targeting domain,^17^ they are better represented by the Bid chimeras than by BH3 peptides. In addition, as tBid (the p15 fragment) becomes intrinsically disordered when bound to membranes,^48, 49, 50, 51^ the final tertiary structures of chimeras and parent BH3-only proteins may be similar. Thus, previous studies with BH3 peptides or truncated BH3-only proteins may have underestimated the ability of the parent BH3-only proteins to activate Bak and Bax, and the Bid chimeras provide an improved means of assessing direct activation.

Our studies indicate that each of the BH3-only proteins, except for Bad and Noxa, have some ability to activate Bak and Bax. Bik, Bmf and Hrk are often considered to be ‘sensitizers' that function only by binding and sequestering prosurvival proteins. However, as our data indicate that they may also activate Bak and Bax, both functions may be important, as deduced for Bim *in vivo*.^29^

Our comparison of Bak and Bax showed that both were activated by the same chimeras, and by the same peptides (Figure 3 and Supplementary Figure 4). This is consistent with liposome studies in which BakΔNΔC and Bax were activated by the same BH3 peptides.^23^ Our findings differ from experiments with permeabilized cells (which may contain prosurvival proteins), where Bak was preferentially activated by Bid, while Bax was preferentially activated by Bim.^25^ Our findings are more consistent with their experiments with isolated mitochondria and liposomes where the preference was less apparent.^25^ Finally, the lack of specificity of BH3-only proteins for activating Bak *versus* Bax, and a role for h0 in activating both Bak and Bax, suggests similar activation sites on both pore-forming proteins. Accordingly, several structures show BH3 peptides bound to the canonical hydrophobic grooves in Bak and Bax.^24, 39, 52^

In conclusion, cells have various means of activating Bak and Bax, as most BH3-only proteins can directly activate Bak and Bax, and others (Bad and Noxa) may indirectly activate the two proteins. Direct activation is greatly enhanced by targeting to mitochondria, suggesting that studies relying on C-terminally truncated BH3-only proteins and BH3 peptides underestimate direct activation by their parent BH3-proteins. The Bid chimeras developed in this study are targeted to membranes to better represent the interactions between BH3-only proteins and Bak and Bax that require a membrane.^17, 33, 43^ Therefore, they are important new tools for *in vitro* studies of Bcl-2 proteins on liposomes, vesicles or mitochondria.

## Materials and Methods

### Generating recombinant proteins and BH3 peptides

Chimeras of human Bid in which the BH3 domain (aa 81–100) was swapped with 20 residues (human Puma 132–151, Bad 105–124, Bik 52–71, Hrk 28–47 and Bax 52–71) or 16 residues (Bid 85–100 replaced with human Bim 147–162, Noxa 24–39, Bmf 170–185 and Bak 73–88) by PCR site-directed mutagenesis. The Bid backbone had a thrombin cleavage site in place of the caspase 8 cleavage site to allow one-step cleavage and purification^30^ and was cloned into pGEX-4T2 to add a C-terminal HA tag. Protein was expressed in *Escherichia coli* BL21 (DE3) induced with 1 mM Isopropyl *β*-D-1-thiogalactopyranoside (IPTG) overnight at 18 °C. Bacteria were lysed in lysis buffer (phosphate buffered saline with 1 mM EDTA, 10 *μ*g/ml aprotinin and 10 *μ*g/ml leupeptin), with 1 mM dithiothreitol (DTT), 0.5 mM phenylmethanesulfonyl fluoride, 266 *μ*g/ml lysozyme and 37 *μ*g/ml DNase I with a homogenizer (EmulsiFlex, Avestin, Ottawa, ON, Canada). The lysate was centrifuged and filtered, before incubation with glutathione beads (GE Healthcare Bio-Sciences AB, Uppsala, Sweden). Washed beads were incubated overnight at 4 °C with thrombin in 50 mM Tris pH 8.0, 150 mM NaCl and 5 mM MgCl_2_. Eluate was further purified by gel filtration (Superdex 75, GE Healthcare Bio-Sciences AB) in Tris buffered saline (TBS; 20 mM Tris pH 8.0 and 150 mM NaCl) with 2 mM DTT. Fractions were aliquoted and stored at −80 °C.

Recombinant full-length human Bcl-x_L_ was cloned into PTYB1 vector to produce a fusion of Bcl-x_L_ with an intein/chitin binding protein, which can be removed to obtain Bcl-x_L_ with vector encoded residues Gly–Ser–Ser at the C terminus. Protein was expressed in ER2566 *E. coli* (New England Biolabs, Ipswich, MA, USA) induced with 0.5 mM IPTG overnight at 18 °C. Bacteria were lysed with a homogenizer in TEN buffer (20 mM Tris pH 8.0, 1 mM EDTA, 500 mM NaCl) with 10 mM MgCl_2_, 25 *μ*g/ml DNase I and protease inhibitor cocktail (Roche, Basel, Switzerland). CHAPS (1%, Sigma-Aldrich, St Louis, MO, USA) was incubated with lysate for 30 min prior to centrifugation and filtration. Clarified lysate was passed through a column with chitin resin, which was then washed thoroughly with 0.2% CHAPS in TEN buffer. On-column cleavage of the intein tag was induced with 50 mM DTT for at least 40 h at 4 °C. Bcl-x_L_ was eluted and further purified by gel filtration (Superdex 200) in TBS. Fractions were concentrated, aliquots flash frozen with liquid nitrogen and stored at −80 °C. BakΔC22-HexHis was produced by the Bax purification protocol described previously.^24^ The expression and purification of human Bax, mouse Mcl-1ΔN151ΔC23, human Bcl-x_L_ΔC25 and human Bcl-2ΔC22 has been described previously.^6, 24^

Human Noxa (residue 1–40) was cloned into pGEX 6P3 vector resulting in a C-terminal GST tag. Protein was expressed in *E. coli* BL21 (DE3) induced with 1 mM IPTG for 3 h at 37 °C. Bacteria were homogenized in TBS-E (TBS with 1 mM EDTA). The lysate was centrifuged and filtered before passing through a GST column. The column was then washed with TBS-E and bound proteins eluted with 10 mM reduced glutathione (in TBS-E) then further purified by gel filtration (Superdex 75) in TBS.

Synthetic peptides based on human BH3 sequences were synthesized by Mimotopes (Notting Hill, VIC, Australia) and purified by reverse-phase HPLC to >90% purity. Full peptide sequences are shown in Supplementary Figure 2. Most peptides have been published previously: Bid (34-mer) in Willis *et al.*^18^ Bim, Noxa, Bad and Hrk in Chen *et al.*^6^ Bak and Bax in Czabotar *et al.*^24^ Noxa mutant peptide sequences are shown in Figure 5c.

### Mitochondrial cytochrome *c* release

MLM were prepared from C57BL/6 wild-type or *Bak*^−/−^ mice as described.^53^ MLM were diluted to 1 mg/ml in MELB (100 mM KCl, 2.5 mM MgCl_2_, 100 mM sucrose, 20 mM HEPES/KOH pH 7.5, 5 mM DTT) supplemented with protease inhibitor cocktail and 4 mg/ml pepstatin A (Sigma-Aldrich). Recombinant full-length Bax, Bid BH3 chimeras, GST-NoxaΔC and BH3 peptides were added as indicated, and samples incubated for 2 h at 37 °C. When chimeras were diluted significantly (e.g., dose–response experiments in Figures 3–5), binding of protein to tubes was minimized by the presence of 1% BSA in diluent (final 0.01 to 0.03% BSA). Similarly, peptides were diluted in 100% DMSO (final 1–3% DMSO on). Supernatant and pellet fractions were separated by centrifugation and analyzed by sodium dodecyl sulfate (SDS)-PAGE and Western blotting for cytochrome *c*.

### SDS-PAGE and Western blotting

SDS-PAGE and Western blotting was performed as described.^54^ Primary antibodies used were anti-cytochrome *c* 7H8.2C12 mouse monoclonal (BD Biosciences, San Jose, CA, USA), anti-HA 16B12 mouse monoclonal (Covance, Princeton, NJ, USA) or 3F10 rat monoclonal (Roche), anti-Mcl-1 19C4-15 rat monoclonal (WEHI mAb Facility, Bundoora, VIC, Australia^55^), anti-Bcl-x rabbit polyclonal (BD Biosciences), anti-Bcl-2 Bcl-2-100 mouse monoclonal (WEHI mAb Facility^56^), anti-Noxa 114C307 mouse monoclonal (Novus Biologicals, Littleton, CO, USA) and anti-Bak 4B5 rat monoclonal (WEHI mAb Facility^57^). Secondary antibodies were horseradish peroxidase-conjugated goat anti-mouse, goat anti-rabbit and goat anti-rat (Southern Biotech, Birmingham, AL, USA).

### Quantitation of cytochrome *c* release and dose–response analysis

Western blot images were quantitated by densitometry with ImageLab 4.1 software (Bio-Rad, Hercules, CA, USA). Percentage cytochrome *c* release was determined independently from the supernatant and pellet densitometry (using Excel for Mac, Microsoft, Redmond, WA, USA) as follows. Percentage release in supernatant was calculated as the ratio of each density to the maximum density in the supernatant blot. The same calculation for pellet blots gave the percentage of cytochrome *c* retained; percent release is then obtained by subtracting from 100%. In cases where the densitometry on the pellet did not drop to zero (i.e., 100% cytochrome *c* is not released) the supernatant percentages for that case were multiplied by densities of sup/(sup+pellet) for the dose corresponding to maximum cytochrome *c* release. The two measures of cytochrome *c* release (from supernatant and pellet) were imported into Prism 6 (Graphpad, La Jolla, CA, USA) and treated as two independent measures. Curves were fitted to the data using ‘log(agonist) *versus* response' non-linear regression. ‘Top' and ‘Bottom' values were fixed at the average minimum and average maximum, respectively, of all blots in that experiment, and the log(EC_50_) and Hill Slope parameters were unconstrained.

### Immunoprecipitation

For immunoprecipitation of Bid BH3 chimeras with prosurvival Bcl-2 proteins, 40 nM Bid chimeras were incubated with 40 nM hBcl-x_L_, hBcl-x_L_ΔC25, mMcl-1ΔN151ΔC23 or hBcl-2ΔC22 in the presence of 1 mg/ml *Bak*^−/−^ MLM. After 1 h at 37 °C, samples were solubilized with 1% digitonin and immunoprecipitated as described^57^ using anti-Mcl-1 14C11 rat monoclonal,^58^ anti-Bcl-x_L_ IC2 rat monoclonal^7^ or anti-Bcl-2 Bcl-2-100 mouse monoclonal antibodies and Protein G Sepharose 4 fast flow (GE Healthcare Bio-Sciences AB).

### Liposome permeabilization assay

Bak-mediated liposome assays were based on previously described methods.^44^ Liposomes were prepared by drying lipid mixes (46% phosphatidylcholine, 25% phosphatidylethanolamine, 11% phosphatidylinositol, 10% phosphatidylserine, 8% cardiolipin and 10% 18:1 DGS-NTA(Ni) 1,2-dioleoyl-sn-glycero-3-[(N-(5-amino-1 carboxypentyl) iminodiacetic acid)succinyl] (nickel salt)) in chloroform and 0.01% butylated hydroxytoluene under N_2_, and then resuspending in liposome buffer (10 mM HEPES pH 7.5 and 135 mM KCl) containing 50 mM 5(6)-carboxy-fluorescein. To remove excess free lipid and collect a uniform size liposome mixture the sample was passed over a polycarbonate membrane filter (diameter 19 mm; pore size 0.1 *μ*m). To remove unincorporated dye, the liposomes were passed through a PD10 desalting column. For each assay, liposomes were used at a final concentration of 2.5 mM and the fluorescence of released self-quenching 5(6)-carboxy-fluorescein measured at an excitation wavelength of 485 nm and emission wavelength of 535 nm.

## Abbreviations

BH3: Bcl-2 homology region 3
HA: haemagglutinin
IVT: *in vitro* translated
MLM: mouse liver mitochondria
MOM: mitochondrial outer membrane
SDS: sodium dodecyl sulfate
SPR: surface plasmon resonance
TBS: Tris buffered saline
