# Supplementary material for: Bid chimeras indicate that most BH3-only proteins can directly activate Bak and Bax, and show no preference for Bak versus Bax
Source: Cell Death Dis. 2015 Apr 23;6(4):e1735–. doi: 10.1038/cddis.2015.105 (PMC4650538; doi:10.1038/cddis.2015.105)
Supplement: Supplementary Figure 1 [file cddis2015105x1.pdf]

Bid chimeras indicate that most BH3-only proteins can directly activate Bak and Bax, and show no preference for Bak versus Bax

Hockings et al.

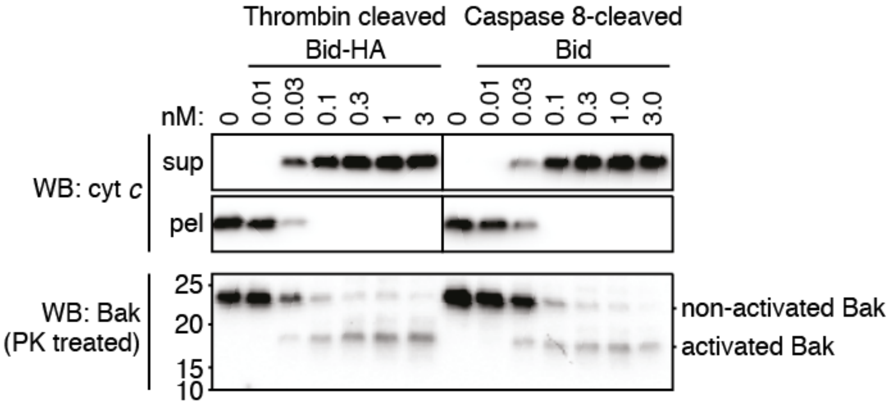

Supplementary Figure 1. A C-terminal HA tag does not alter Bid function.
Wild type MLM were incubated with two forms of Bid as indicated, and assessed for cytochrome *c* release and for Bak conformation change by susceptibility to proteinase K (PK)(Ma et al, *Cell Death Differ* 2014; 21: 1925-1935). Note that thrombin-cleaved Bid-HA and caspase-8-cleaved Bid activated Bak (convert to a PK-sensitive conformation) and induced cytochrome *c* release with the same potency. Blots are representative of two independent experiments.

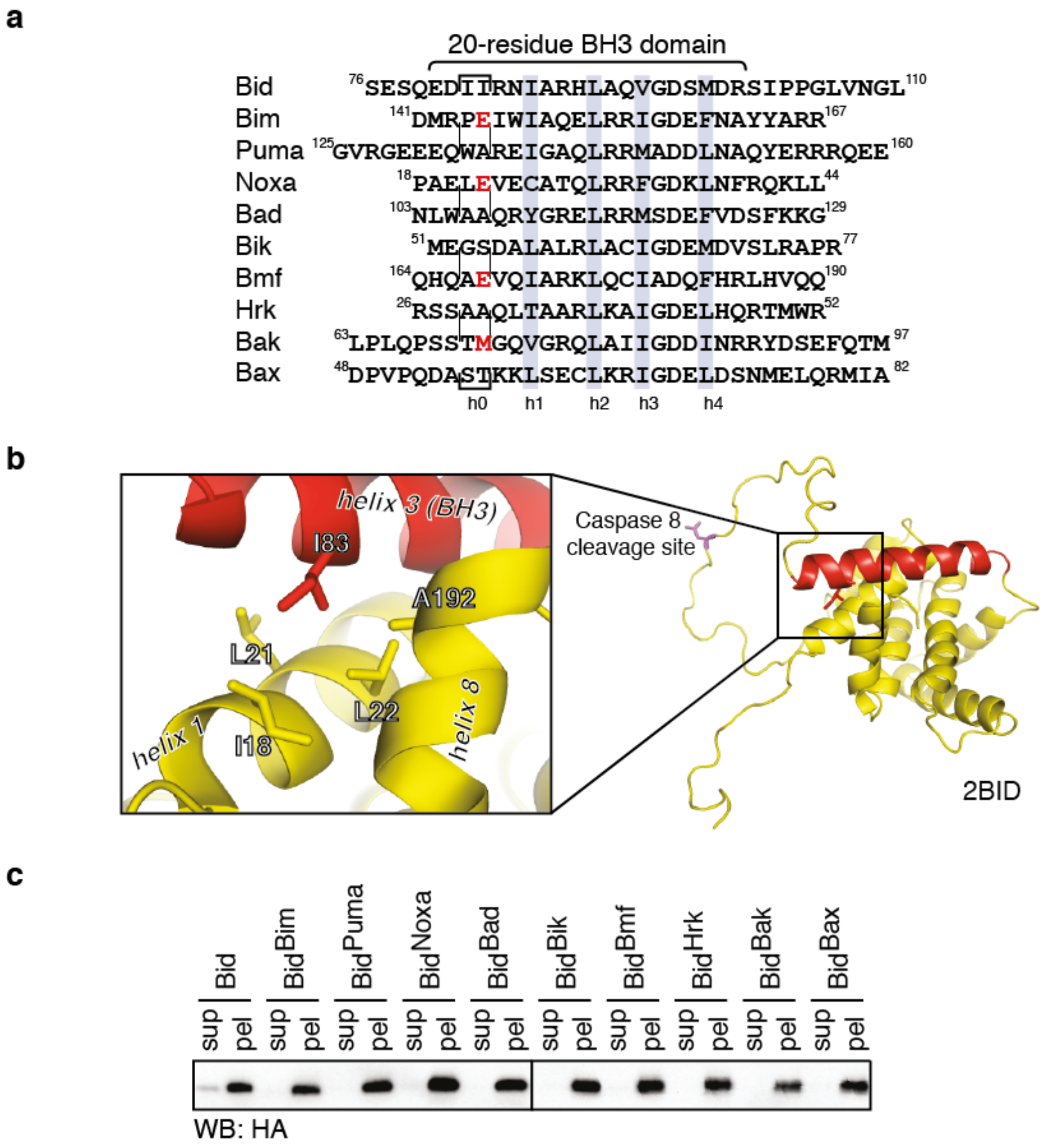

Supplementary Figure 2. Bim, Noxa, Bmf and Bak BH3 domains contain a residue that may destabilize the Bid chimera.

(**a**) Sequence alignment of the BH3 peptides used in this study, highlighting the h0-h4 positions in the BH3 domain. The presence of glutamate or methionine at the equivalent Bid I83 position is highlighted (red). Note that when generated as 20-residue swaps in Bid, the resulting four Bid chimeras (Bid^{Bim}, Bid^{Noxa}, Bid^{Bmf} and Bid^{Bak}) were unstable (not shown).

(**b**) Bid I83 contacts hydrophobic residues in helices 1, 3 and 8. Cartoon representation of human Bid (Chou et al, *Cell* 1999; 96: 615-624) with sidechains shown for I83 and contacting hydrophobic residues.

(**c**) Bid chimeras efficiently target membranes. The indicated Bid chimeras (5 nM, diluted in 1% BSA) were incubated with $Bak^{-/-}$ MLM for 1 h at 37 °C. Supernatant (sup) and pellet (pel) fractions were Western blotted for HA (HA-tagged chimeras). Blots are representative of two independent experiments.

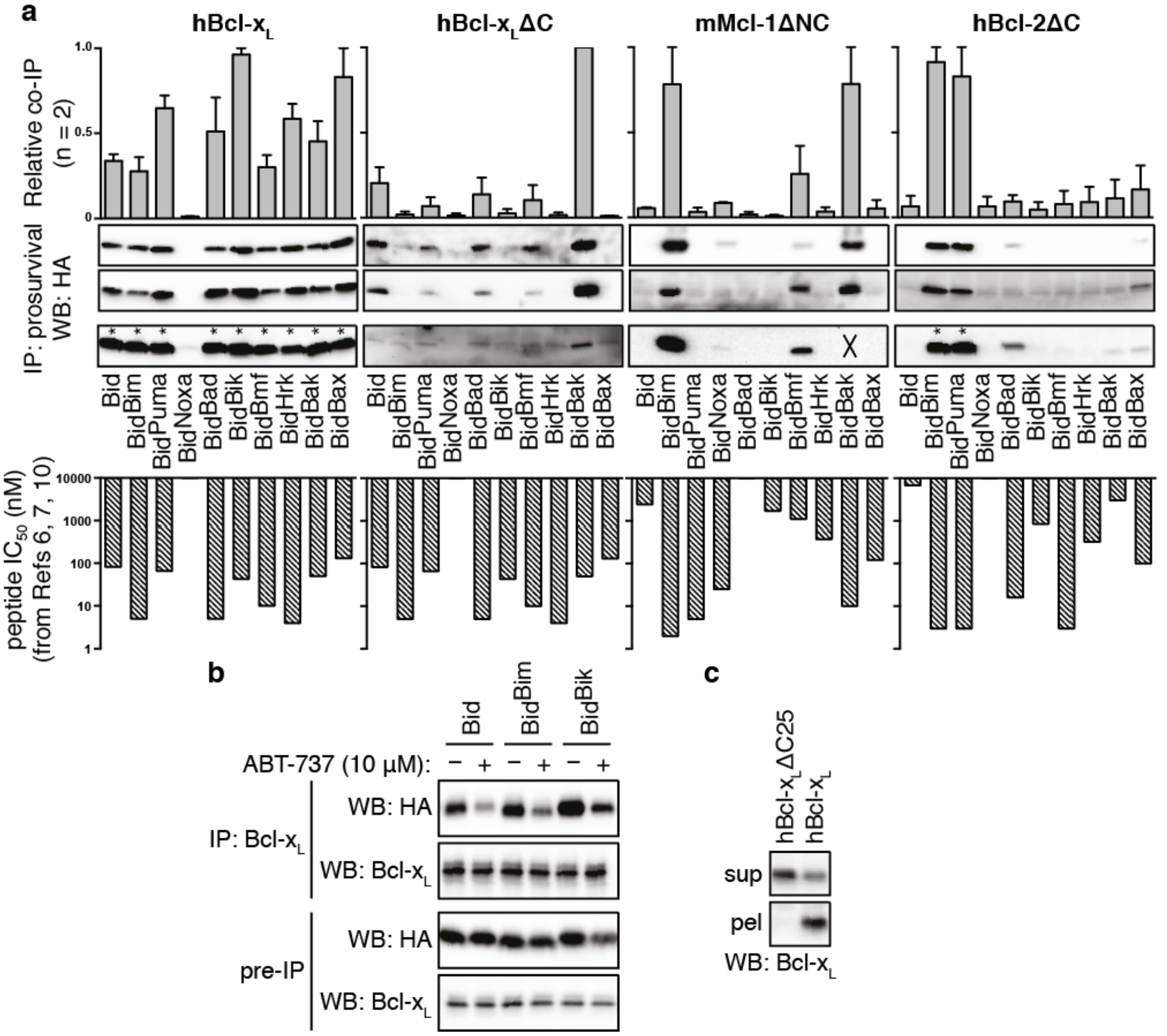

Supplementary Figure 3. Bid chimeras bind to the canonical hydrophobic groove in full-length Bcl-x_L.

(**a**) Quantitation of Bid chimera binding to prosurvival proteins (related to Figure 2). Co-immunoprecipitation of chimeras with the indicated prosurvival proteins is shown for three separate experiments including those in Figure 2 (*upper blots*). Upper bar charts show the co-IP of each chimera (mean and SEM for the top two blots) relative to the strongest band on each blot. While the lower blots reflect the same pattern of co-IP, they were not included in the bar chart data due to overexposed bands (*) or missing sample (X). Lower bar charts show peptide binding affinities measured by SPR as in Figure 2.

(**b**) The BH3 mimetic ABT-737 prevents Bid chimeras binding to Bcl-x_L. Bid, Bid^{Bim} and Bid^{Bik} (40 nM, diluted in 1% BSA) were incubated with full-length hBcl-x_L (40 nM) and *Bak*$^{-/-}$ MLM, in the presence or absence of ABT-737 (10 μM). Samples were immunoprecipitated (IP) for Bcl-x_L as in Figure 2 and Western blotted (WB) for HA (HA-tagged chimeras) or Bcl-x_L.

(**c**) Full-length Bcl-x_L associates with the membrane fraction while truncated Bcl-x_L remains largely in the supernatant. Bcl-x_L or Bcl-x_LΔC (40 nM, diluted in 1% BSA) were incubated with *Bak*$^{-/-}$ MLM for 2 h at 37 ^{o}C and separated into supernatant (sup) and pellet (pel) and Western blotted (WB) for Bcl-x_L.

Blots for (b) and (c) are representative of at least two independent experiments.

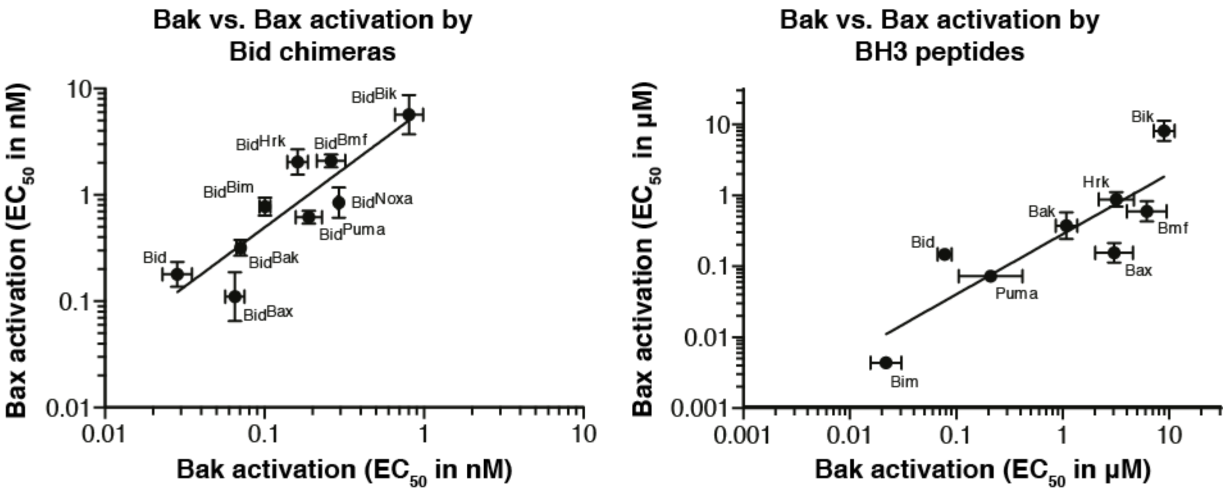

Supplementary Figure 4. Chimeras and peptides show no apparent preference for activating Bak *versus* Bax.

EC_{50} values from Figure 3b were plotted to compare the ability of each chimera or peptide to activate Bak (*x*-axis) and Bax (*y*-axis), with corresponding error bars (SEM) shown. Correlation was calculated using Prism 6 (Graphpad) from logarithms of EC_{50} values on a linear scale, and a logarithmic scale applied for ease of reading. $r^2 = 0.76$ for chimeras and $r^2 = 0.74$ for peptides.

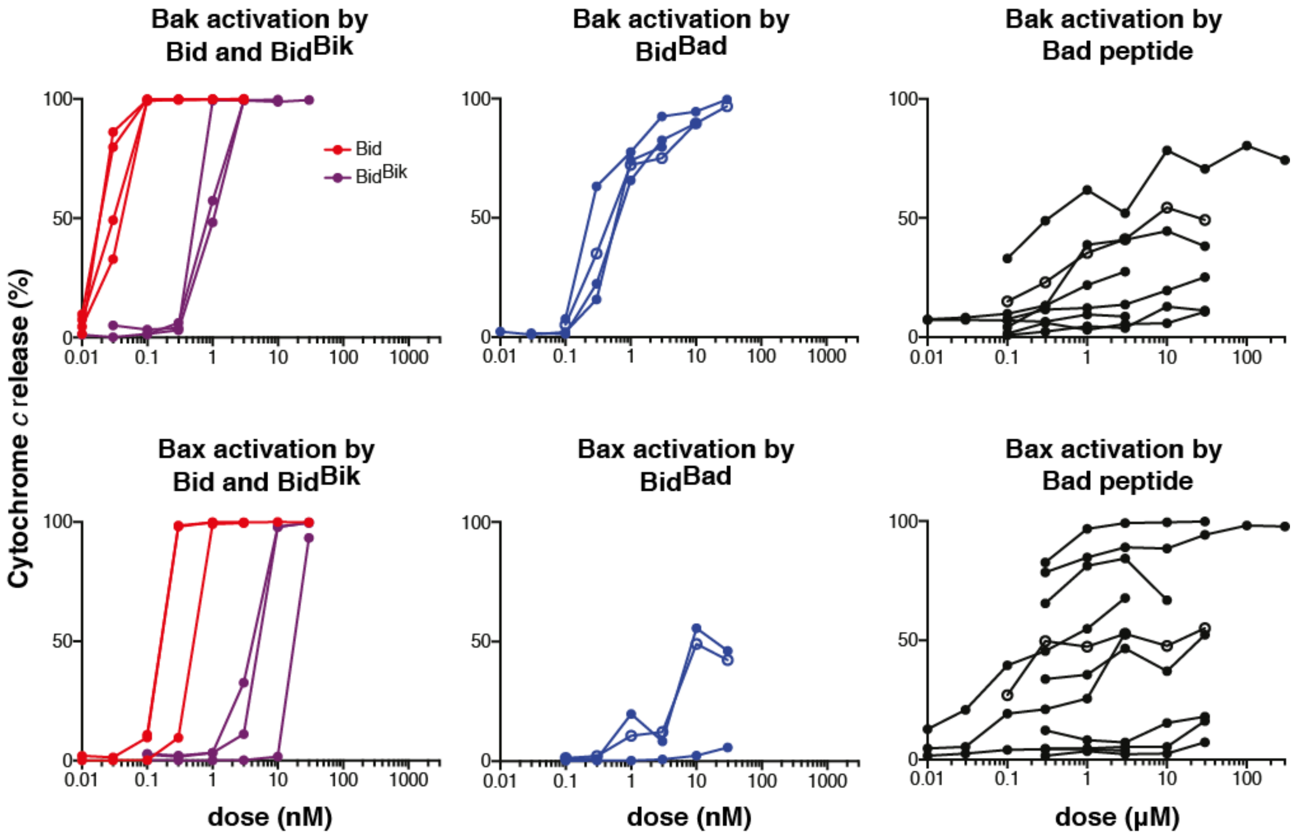

Supplementary Figure 5. Bid^{Bad} and Bad BH3 peptide initiated cytochrome *c* release is often incomplete.

Repeat experiments for MLM cytochrome *c* release by the Bid^{Bad} chimera and Bad BH3 peptide are shown, as well as the Bid and Bid^{Bik} data from Figure 3b. The Bid^{Bad} chimera and Bad BH3 data from Figure 4 are the traces with open circles. Note that release initiated by Bid and Bid^{Bik} becomes complete when concentration is increased 3- to 10-fold, whereas release initiated by Bid^{Bad} and Bad peptide is more graded and often incomplete.
